# Compiling video cases to support PD facilitators in noticing productive teacher learning

**DOI:** 10.1186/s40594-018-0147-y

**Published:** 2018-11-28

**Authors:** Sven Schueler, Bettina Roesken-Winter

**Affiliations:** Humboldt-Universität zu Berlin, Unter den Linden 6, 10099 Berlin, Germany

**Keywords:** Facilitation, Mathematic, Professional development, Video cases, Teacher learning

## Abstract

**Background:**

Research studies on facilitating professional development describe the knowledge and skills facilitators need to effectively attend to teachers’ learning processes. In this context, some studies gave rise to the question how to design learning opportunities to prepare facilitators to support teachers’ learning during professional development. The approaches share putting mathematics specialized content knowledge, pedagogical content knowledge on the professional development level and facilitation moves in the center. Research findings also highlight the role of video-based learning opportunities to qualify facilitators respectively. In particular, structured approaches that guide facilitators noticing toward advancing teacher learning have to be proven effective. Although research highlights using video-based material as a training tool for facilitators, what exactly the learning opportunities should consist of is up for discussion. In this article, we report on a validation study concerned with designing video cases, taken from a videotaped teacher professional development. We explored how eight experienced facilitators perceived the representativeness and the quality of the video cases for using them in facilitator professional development with respect to specialized content knowledge, pedagogical content knowledge on the professional development level, and facilitation moves. Additionally, we investigated what facilitation moves are noticeable in the video cases, and what noticing prompts can enhance these learning opportunities.

**Results:**

Our results indicate that all video cases present vital opportunities to discuss how to support teacher learning in terms of specialized content knowledge, pedagogical content knowledge on the professional development level and facilitation moves. Our analyses provide insights into facilitators thinking processes, thus enriching their ratings by ideas and concerns considering the final implementation of the video cases in facilitator professional development. On the basis of our validation study, we were able to carefully check the learning opportunities that the chosen video cases can provide for facilitator professional development.

**Conclusions:**

This paper provides information on how to design video-based learning opportunities for facilitator professional development. Although our results are of course bound to the six video cases we selected, our approach can be transferred to other contexts. The procedure of compiling the video cases and validating them takes up the most prominent research findings on designing facilitator professional development and pays careful attention to experiences facilitators thinking. We exemplarily show how the empirical evidence can be used to prepare facilitator PD.

## Introduction

“So, I could toss one coin four times, or four coins at once, right? Does this make any difference for the probability of obtaining heads or tails?” Jane, a primary teacher in her fifth year of teaching mathematics, asked this question in a mathematics professional development (PD) course on teaching probability and statistics. The question arose after the PD facilitator introduced a task dealing with determining the probability distribution of heads and tails when a coin is flipped four times. The facilitator noticed Jane’s indecision, but decided to leave the question unanswered. Instead of responding directly, she asked the teachers to think about it while solving the task. The mathematical background addressed by the question was then reflected upon in an in-depth discussion. Thus, this small incident turned out to result in a fruitful learning opportunity for all of the teachers in the PD.

PD facilitators encounter such situations on a regular basis. Just as teachers in the classroom, they have to constantly anticipate the mathematical relevance of a teacher statement and need to decide in-the-moment how to proceed. The in-the-moment decision-making includes reflections such as the following: *Should I just give Jane an answer right away*? *What mathematics content am I going to address next*? *How can I value Jane*’*s question positively to not make her feel like she does not know much about probability and statistics*? These are just some of the possible decisions mathematics PD facilitators might keep in mind when leading a PD course with the explicit goal to support teachers’ mathematics learning. The facilitator in our example has extensive experience in providing PD, and was thus able to allow for a little detour to clarify the underlying mathematical concept. The question remains, however, how would novice facilitators act in such a situation? And more importantly, how can they gain such an expertise that normally develops over time and with practice? In our research project, we build on such situations that are crucial for teacher learning and question how PD facilitators can enhance their capability to respond adequately. Particularly, we scrutinize how material for “facilitator PD” can be designed to enable facilitators-in-training to later provide robust opportunities for teachers’ mathematical learning in PD courses they facilitate on their own.

As regards the general composition of PD courses, the last two decades have contributed research findings on the design and implementation of effective PD programs (Timperley et al. 2007; Desimone 2009). Specific research on PD facilitation is still an emerging field and Borko et al. (2014b) underlined that “especially critical is research on the knowledge and practices necessary to be an effective leader of mathematics PD” (p. 150). Research studies have shown that novice facilitators may possess both profound mathematics content knowledge and pedagogical content knowledge, but often struggle with effectively supporting teacher learning (Borko et al. 2014b; Even 2014; Borko et al. 2014a). As a quintessence “just like classroom teachers, facilitators need focused support prior to using a formal PD program to ensure that they can effectively pursue opportunities to unpack and build on teachers’ ideas in line with the program’s goals (Remillard and Geist 2002)” (Jacobs et al. 2017, p. 3).

Going back to the example mentioned in the beginning, we question *what* specific support facilitators need to be able to notice productive teacher learning. A promising practice-based approach lies in using *video cases* to provide active learning opportunities*.* On the teacher PD level, many studies have highlighted the positive effect of using video cases to enhance teachers’ noticing of essential aspects of practice (e.g., Beisiegel et al. 2018). Facilitators who obtained the possibility to discuss their role in video clubs could also improve their noticing and were found to be more supportive as teacher PD leaders (e.g., González et al. 2016). Nevertheless, using video cases to prepare facilitators for their crucial role involves careful and advanced designing of such learning opportunities (Lesseig et al. 2016).

In the present study, we draw on the aforementioned findings and report on validating *video*-*based learning material* for facilitator PD. Our particular focus is on providing learning opportunities for facilitators to support them in noticing productive teacher learning based on their knowledge and skills. In our validation study, experienced facilitators carefully checked the developed video cases to serve their function.

In chapter 2, we situate our study against the background of the current state of research. First, we discuss what knowledge facilitators need to support teacher learning and clarify the constructs addressed. Second, we pay specific attention to related facilitation moves and available frameworks. Third, we discuss design principles for facilitator PD to inform our validation approach. In chapter 3, we concretize the aim of our study in light of the theoretical background and formulate our research questions. Information on methodological considerations of the validation is then provided in chapter 4. In chapter 5, we present the results of our study. We end by discussing our findings in chapter 6 and by illustrating implications for our design of facilitator PD. Furthermore, we suggest future directions of video-based facilitator PD design and research.

## Theoretical background

PD facilitation is a complex endeavor. The success of a PD program is strongly tied to the facilitator’s ability to guide teachers’ learning toward the intended goals. When conducting a PD course “facilitators, like teachers, must make continual, in-the-moment decisions that take into account the interplay between their PD program, the participants, and the situational context” (Jacobs et al. 2017, p. 11). In our validation study, we combine the following perspectives: From a content level, we question what *knowledge* and *skills* constitute PD facilitation. Our literature review first addresses what knowledge facets are relevant in the context of PD facilitation. Second, we consider facilitation moves frameworks that pay attention to practices that help teachers achieve the learning goals during PD. From a methodological level, we scrutinize how prospective facilitators can learn such practices and how they can enhance their noticing of crucial incidents during PD. We then report on design principles to ensure that facilitator PD can provide the necessary learning opportunities to attain the *knowledge* and *skills* needed. Against this theoretical background, we compiled video cases as learning opportunities for facilitators. In a validation study, we then checked whether the theoretical considerations are reflected in our design.

### Facilitator knowledge and skills

Orchestrating effective teacher learning during PD demands, according to Borko (2004), the following three practices: engaging teachers in productive mathematical work, leading discussions about student reasoning and instructional practice, and building a professional learning community. Based on this list, it becomes apparent that facilitating PD demands a profound knowledge of the mathematical content relevant in the PD context. On the teacher level, many research contributions have informed the potential facets that comprise teachers’ professional knowledge (Ball et al. 2008). On the facilitator level, there is still discussion concerning what knowledge facets are important. Following Jacobs et al. (2017), who drew on Ball et al.’s (2008) construct of mathematical knowledge for teaching, we consider *mathematical knowledge for facilitating PD* to comprise of specialized content knowledge (SCK) and pedagogical content knowledge (PCK). SCK comprises “a sophisticated understanding of the mathematical concepts and relationships intended to be covered during the PD” (Jacobs et al. 2017, p. 3). Accordingly, Lesseig et al. (2016) emphasized the role of SCK for strategically selecting, sequencing, and pursuing key mathematical connections for teachers’ learning of mathematics. PCK includes a facilitator’s ability to “engage teachers in purposeful activities and conversations about those mathematical concepts and relationships and to help teachers gain a better understanding of how students are likely to approach related tasks” (Jacobs et al. 2017, p. 3). In order to highlight that the notion of PCK was adapted to the facilitator level, we use the term *PD*-*PCK* in the following.

Given a particular topic such as *probability and statistics* in PD, the facilitator needs to plan the order of the different sub-themes, and to choose material and resources. Facilitators also decide how to involve teachers as learners of the mathematical content and what aspects to discuss from a classroom perspective while drawing on exemplary student work. When planning and conducting a PD course, facilitators thus constantly draw on their SCK and PD-PCK. Van Es and Sherin (2017) compared such considerations with preparing teaching in the classroom: “Similar to teachers’ work with curriculum materials, facilitators need to understand in a detailed way the purpose of the professional development materials they use and what the program is trying to achieve and why (Remillard, 2005)” (p. 1). As Borko et al. (2014a, b) demonstrated, novice PD facilitators particularly struggle with supporting teacher discussion in terms of SCK.

Summing up, one essential aim of facilitator PD lies in providing learning opportunities on the SCK and PD-PCK level. In focus are aspects that aim at reaching the specific learning goals on the teacher PD level. In our study, these knowledge facets are one essential category for designing facilitator PD based on video cases and constitute one content dimension of our validation study.

### Facilitation moves

Another line of research is concerned with elaborating how *knowing* becomes *acting* and what moves characterize effective facilitation. González et al. (2016) defined facilitation as the “performance of specific moves according to the core activities in the professional development” (p. 448). Different frameworks have emerged that describe such facilitation moves. For facilitating teachers’ analysis of video cases, van Es et al. (2014) contributed a framework consisting of four main categories and several sub-categories (see Table 1).

**Table 1:** Van Es et al.’s (2014) framework for facilitating of video-based discussions

Category	Facilitation move
Orienting group to the video analysis task	Launching
	Contextualizing
	Highlighting
Sustaining an inquiry stance	Lifting up
	Pressing
	Offering an explanation
	Countering
	Clarifying
Maintaining a focus on the video and the mathematics	Redirecting
	Pointing to evidence
	Connecting ideas
Supporting group collaboration	Standing back
	Distributing participation
	Validating participant ideas

The framework comprises 14 different moves that are assigned to the 4 main categories “orienting group to the video analysis task,” “sustaining an inquiry stance,” “maintaining a focus on the video and the mathematics,” and “supporting group collaboration.” Van Es et al. (2014) described the facilitation moves in detail. For instance, *lifting up* refers to “identifying an important idea that a participant raised in the discussion for further discussion” or *countering* to “offering an alternative point of view”. Additionally, to carefully explain the different facilitation moves, the authors exemplified what facilitator statements or prompts can be classified under the different facilitation moves. For instance, the prompt “you said there was a lot she had to do there, can you piece apart for me all the things you think she had to do?” was classified as a *pressing* facilitation move. The framework was especially constructed to capture the facilitation of video-based discussion. González et al. (2016) extended this framework to be applicable to PD that not only includes videos but also animations. As a result, these authors partly modified the initial framework (see Table 2).

**Table 2:** Effective facilitation moves for combined PD according to González et al. (2016)

Category	Move	Description
Orienting the group to the task analysis	Launching	Ask general questions to help begin the discussion.
	Contextualizing	Share background knowledge that could be useful.
	*Moving along* ^a^	*Transition to a new activity in the professional development*.
Sustaining an inquiry stance	Highlighting	Emphasize an idea the teachers missed *in the representation*.
	Lifting up	Emphasize an idea a teacher proposed in the discussion.
	Pressing	Ask a teacher for more details about a comment.
	Clarifying	Restate a participant idea to help make its meaning clear.
	Offering an explanation	Make a substantive comment about *the representation, including examples* from own classroom.
	Countering	Provide an alternative to a participant idea.
Maintaining a focus on the professional development and the mathematics	Redirecting	*Refocus the group on goals of the professional development*.
	Pointing to evidence	Comment on the representation and support with evidence.
	Connecting ideas	Link ideas between *participants or parts of the session*.
Supporting group collaboration	Standing back	Allow the members of the professional development to talk.
	Distributing participation	Encourage different teachers to contribute.
	Validating participant ideas	Give positive feedback to the teachers.
Other moves	None	*Coordinate details of the session that are not relevant to the discussion*.

^a^González et al. (2016) originally noted new moves or descriptions of new moves in italic

In comparison to the original framework, González et al. (2016) changed the main category of “maintaining a focus on the video and the mathematics” to “maintaining a focus on the professional development and the mathematics”. They also added one main category, and one move. Additionally, the authors broadened the definition of two of the moves, namely *connecting ideas* and *offering explanations*. Regarding the relevance of the framework, González et al. (2016) underlined that becoming a facilitator entails “developing an understanding of facilitation moves that can promote teacher learning in professional development […]” (p. 462).

Although the modified framework was also developed in a specific context, the authors emphasized that their findings do not show significant differences in the moves for both artifacts videos and animations. This outcome is promising with respect to generally describing facilitation moves for promoting teacher learning in PD. In our study, we thus consider this facilitation move framework as essential category for designing facilitator PD based on video cases. Ultimately, the facilitation move framework represents the second content dimension of our validation study.

### Designing facilitator PD

Facilitators “need opportunities to notice facilitation skills and resources at play in PD. In particular, they need to notice the entailments of facilitation such as how a facilitator pursues teacher learning goals within the complex interactional work of PD” (Lesseig et al. 2016, p. 4). The relevance of such learning opportunities within facilitator PD is no longer debated. However, how to design PD for such learning essentials is only partly answered in the research literature. As a starting point, we can draw on the following design principles for facilitator PD by Lesseig et al. (2016): (1) center leaders’ mathematical work on clear learning goals for teachers, (2) design tasks for leaders that explicitly target SCK goals, and (3) provide video cases (or other artifacts of practice) that exemplify instances when mathematical goals are being pursued and facilitate analysis of the video case to make the work of the PD leader explicit. When critically reviewing the different conceptualizations of facilitator PD they tested, Lesseig et al. (2016) underlined that they initially relied too much on the video cases themselves. They found focusing on the SCK aspects shown in the video cases essential to advance teacher learning. In addition, they showed that facilitators need help in identifying SCK goals and in consequently keeping the focus on the mathematical aspects during PD.

Findings from lesson analysis studies confirmed that guiding reflections help to focus discussions on essential aspects. Santagata and Angelici (2010), for instance, revealed that scaffolding reflections by prompts such as “What questions does Ms. Thompson ask the students? Why do you think she chose those questions?” helped pre-service teachers to keep the focus on essential teaching aspects. Sherin and van Es (2005) also included specific questions in their use of video to foster pre-service teachers’ noticing: “What do you notice? What’s your evidence? What’s your interpretation of what took place?” The prompts were specifically used to guide teachers’ noticing, which consists of selective attention, knowledge-based reasoning, and decision-making.

In our final design of the video cases, we need to ensure that the focus of the facilitators’ discussions remains on those aspects of SCK, PD-PCK, and on the facilitation moves we want to address. Therefore, we opt for using the video cases for the facilitator PD in a structured way and add prompts to guide facilitators’ noticing toward these aspects. The research findings discussed above also emphasize that design decisions have to be carefully checked to ensure they serve their aim. In this article, we thus report on a validation study with a twofold purpose: First, we involved experienced facilitators in a rating study in order to gain evidence of validity regarding the content of the developed video cases concerning SCK, PD-PCK, and facilitation moves. Second, we requested experts to provide alternative facilitation moves and noticing prompts for each of the video cases to help facilitators to focus on these aspects and to enhance their learning.

## The present study and research questions

As the literature review revealed, supporting teacher learning toward the intended goals in a PD course is a challenging endeavor. Facilitators need strong SCK, PD-PCK, and should be able to perform specific facilitation moves to advance teachers’ mathematics learning. In chapter two, we also presented three design principles for facilitator PD. In our study, we take up these suggestions and aim at designing a specific element of facilitator PD. We compile video cases from teacher PD to inform facilitator PD, and pay attention to the following aspects: for each of the cases, we make the teacher learning goals explicit, and provide background information on both the material used and the solutions the teachers generated (design principle 1). In consideration of design principles 2 and 3, we will add prompts to each video case to ensure facilitators’ focus on noticing SCK, PD-PCK, and facilitation moves in the final design.

When designing video cases of PD to aid leader noticing of the described knowledge facets and reflecting upon possible moves, we need to validate that the chosen scenes are suitable to reach these aims. The purpose of our validation study, immersing facilitators with different degrees of experiences, is twofold: On the one hand, we check the video cases for their content validity. We seek to explore whether the video cases allow for intense discussion on SCK, PD-PCK aspects, and facilitation moves. On the other hand, we ask for alternative facilitation moves and noticing prompts, revealing interpretive arguments that specify the proposed interpretations (cf. Kane 2006). Later, we will finally design the structured video cases and propose prompts that the “facilitator” of the facilitator PD can use to elicit facilitators’ noticing. However, in the study at hand, we limit ourselves to report on the validation process. In the discussion, we reflect upon implications for the final design.

In the following, we provide further information on how we prepared the video cases and how we compiled the rating. Additionally, we exemplarily describe one video case in detail by outlining the context and setting as well as the SCK, PD-PCK, and facilitation moves involved.

### Preparing video-cases

In late 2017, we videotaped 3 days of a PD course for primary mathematics teachers in the federal state of Berlin, Germany. The PD course was practice-based and focused on crucial features of teachers’ SCK and PCK in the domain of probability and statistics. The overall setting of the program required participating teachers to meet four times in one semester for collaborative learning with an experienced PD facilitator. Additionally, teachers were given tasks to build into their instructional practice for the time in between the meetings.

In total, we recorded and prepared approximately 20 h of video material for further analysis. After an initial viewing of the entirety of the video material, we developed a coding system to describe specific sequences, showing crucial incidents of teachers’ learning of probability and statistics in the PD setting.

In order to structure the coding process, we delineated units of meaning relevant to the research questions (cf. Cohen et al. 2007). That led to dividing the video material of each PD day into segments, showing different phases, topics, or teacher learning goals. In our study, we refer to these units as idea units. For example, one idea unit deals with the facilitator’s instruction of cumulative frequency. The next idea unit covers the teachers working on a task concerned with the implementation of the concept of cumulative frequency in their classes. For each idea unit, we coded beginning, end, and duration, followed by a short description of the content of the PD course, the teacher learning activities, and the facilitation moves shown in the scene.

For each idea unit, we then decided whether to include or exclude the scene as a video case for the rating study. The goal of this process was to provide video cases, which depict how the facilitator supports teachers’ mathematics learning in the PD course. For each idea unit, the decision to include or exclude the scene is based on the *mathematics content* and *four exclusion criteria*. In terms of mathematics content, we tried to cover all relevant topics of the PD program: chance and probability, descriptive statistics, and combinatorics. From the literature on teachers’ learning of probability and statistics, we know that especially chance, combinatorics, and descriptive statistics are challenging for teachers, as many misconceptions interfere with the mathematical concepts (cf. Shaughnessy 1993). In the process of structuring the video material, we thus paid attention to incidents showing common misconceptions in probability and statistics. The first exclusion criterion is SCK (*EC1*)*.* The idea unit treats the understanding of key mathematical concepts and relationships. Hence, scenes showing teachers’ learning not related to SCK were excluded. The second criterion for exclusion is PD-PCK (*EC2*)*.* The idea units depict vital opportunities for teachers to actively engage in learning. Scenes only showing the facilitator explaining mathematics concepts without further interaction or instruction were excluded. Third, we coded scenes indicative of *facilitation moves* (*EC3*). This criterion describes scenes that show facilitation moves to advance teachers’ learning of mathematics. We excluded, for example, scenes of the PD course showing participants working on tasks individually. The last criterion considers the *quality of video and audio* (*EC4*). To ensure high quality of our video data, we excluded scenes that were difficult to understand.

Two researchers pre-structured and coded the video material based on the four criteria. Disagreements were mutually discussed until consensus about including or excluding the respective scene was reached. As a result, we selected six video cases, lasting between 3 and 8 min, for further rating. An overview of the video cases that were chosen for the rating is given in Table 3. The teacher learning goals are briefly outlined for each video case.

**Table 3:** Overview of the selected video cases

Video case	PD topic	Duration	Code	Teacher learning goals
“Representing characteristics”	Descriptive statistics	05:58 min	VS_DS1	Teachers know the characteristics of different representations of data sets.
				Teachers can deal with discordant values and inconclusive data sets.
“Law of large numbers”	Descriptive statistics	02:49 min	VS_DS2	Teachers understand the concept of the law of large numbers.
				Teachers know about the representativeness of large data.
“Fitting representations”	Descriptive statistics	08:04 min	VS_DS3	Teachers know a variety of representations for statistical data.
				Teachers can assign correct representations to the characteristics of the data set.
“Symmetry of random events”	Chance and probability	03:17 min	VS_CP	Teachers are familiar with the symmetry of the binomial coefficient.
				Teachers compute the probability of basic random events.
“The bonbon-task”	Combinatorics	04:08 min	VS_CO1	Teachers know basic combinatorial selection processes.
				Teachers can select the fitting combinatorial model for a given task.
“The tower-task”	Combinatorics	05:12 min	VS_CO2	Teachers know the mathematical concept of factorial.
				Teachers can select the fitting combinatorial model for a given task.

In the following, we describe one video case in detail. First, we outline the *context and setting* to give some background information on learning goals as well as the tasks and material used in the PD course. Second, we exemplify how the video cases address the relevant constructs of SCK, PD-PCK, and facilitation moves.

### Video case example: “representing characteristics”

#### Context and setting

The video case “Representing characteristics” was recorded on the first day of the PD course. The topic of the first half of the PD course was concerned with different types of data sets, methods of data collection, and data representation. The second half-day focused on statistic parameters to evaluate statements related to data collections. Furthermore, the implementation of statistics in school curricula was addressed and discussed. Prior to the content depicted in video case 1, the participating teachers worked in groups on the following tasks:

a) Plan and conduct a data collection for a topic of your choice with the participants of this PD course. Work in groups of three or four.

b) Illustrate your data collection for presentation and give reasons for the chosen topic and illustration.

The video case shows two groups of teachers presenting their results to the above tasks. It also shows the PD facilitator taking notes on a flipchart during the teachers’ presentations. The video case ends with the PD facilitator sharing her notes with the teachers and summarizing the teachers’ learning processes.

#### SCK

VS_DS1 is concerned with key concepts of descriptive statistics: evaluating different types of data, dealing with differences in characteristics, and adequate data representations. The two groups of teachers presenting their results focused their data collection on the characteristics “shoe size” and “grade level the participants teach.” In their presentations, teachers struggled with the question whether it makes a difference to use a distinct characteristic or one that allows multiple possible answers. Illustrations used include a bar graph for “shoe size” and a table for “grade level the participants teach.” Referring to the characteristic shoe size, one teacher mentioned that he expected a bell-shaped curve, making connections to the mathematics concept of a Gaussian distribution. Furthermore, the teachers discussed problems with discordant values such as very small or very large shoe sizes.

#### PD-PCK

Involving teachers in these tasks enabled them to independently plan, implement, and illustrate a data collection. Based on the examples that were presented, the teachers were asked to discuss characteristic features and pitfalls of illustrating different data sets. The teachers then reflected on possible student solutions on the basis of their own contributions and discussed possible reactions in depth to advance the learning of their students.

##### Facilitation moves

At the beginning of the video case, the PD facilitator summarized the task the teachers were working on and then asked the first group to present their results. The PD facilitator first showed the bar graph the group had developed to the other teachers, but then she decided to take notes on a different flip chart. The notes the PD facilitator had been taking were not visible for the teachers. Then, the second group presented their data collection and illustration. The PD facilitator kept making notes without interrupting the second presentation. In the last part of the video case, the PD facilitator summarized the teachers’ results and shared her notes with the groups, which concerned: *posing precise questions when planning a data collection*, *specifics of distinct characteristics and scale* and *discordant values*. Furthermore, the PD facilitator explained to the teachers what crucial incidents occurred and how they play a role when teaching data collection and illustration to students in class. She referred to similar problems and questions teachers might encounter when discussing results with their students, hence connecting the PD and the classroom level. At the end of the video case, the PD facilitator connected the notes she made to the content of the PD program, highlighting the importance of teachers’ learning goals. In Table 4, we assigned the different facilitation moves shown in the video case to the categories González et al. (2016) used in their framework for facilitator PD.

**Table 4:** Example of assigning facilitation moves to video case “Representing characteristics”

Speaker	[Setting] Turn	Facilitation move
Facilitator	For the last minutes, you were working together on the task and you had to find an agreement concerning the specific questions in which you are interested.	Contextualizing
Facilitator	Please explain to the other participants why you chose the topic for your data collection and what are your results.	Launching
Group 1	[Presents their results of the task to plan, conduct and illustrate a data collection. Characteristic: shoe-size, Illustration: bar graph]	
Facilitator	[Starts to take notes during the presentation on a flipchart.]	Lifting up (implicit)
	Please do not feel interrupted. I just got to capture a few remarks we might discuss later on.	
Group 1	[Presentation ends with the issues distinct characteristics and discordant values.]	
Facilitator	Do the other participants have any questions for the group? Did you have any problems with the task?	Clarifying
Group 2	[Presents their results of the task to plan, conduct and illustrate a data collection. Characteristic: grade level participants teach, Illustration: table]	
Facilitator	[Stands back and takes notes during the presentation on a flipchart.]	Lifting up (implicit)
Group 2	[Presentation ends] We had the problem to pose a precise question to our data collection. Our results show that the characteristic we chose is open for multiple answers.	
Facilitator	I see you wrote down that problem already on your chart. There have been questions from the other participants. It seems that for many this was not clear.	Highlighting
Facilitator	[After the presentations of all groups.] Thank you for your contributions. I have tried to capture some key aspects that might be relevant for our PD learning to come and your teaching as well.	Connecting ideas
Facilitator	You mentioned earlier, the problem of precise questions in a data collection. This will be one of our next topics.	Lifting up
Facilitator	You have talked specifically about data illustration and the problem with discordant values. Using an interval was another issue. What do I mean, talking about an interval?	Lifting up
Teacher	We have discussed this in the context of the characteristic shoe size. It means the notion of a distance.	
Facilitator	You are right. So, we can use a distinct characteristic, but we can also connect a distinct characteristic with an interval scale.	Validating participant ideas
		Offering an explanation

Information on what happens during the video-case is described in []

In terms of the framework by González et al. (2016), we attained a description of the video case revealing different facilitation moves that characterize the interaction between the facilitator and the teachers. The observed facilitation moves can be assigned to all four main categories: *orienting the group to the task analysis*, *sustaining an inquiry stance*, *maintaining a focus on the professional development and the mathematics*, and *supporting group collaboration.* Thus, the video case allows for discussing the relevant facilitation moves.

However, decisive for designing facilitator PD is not what the researchers who possess a broad and thorough understanding of the scenes taken from teacher PD can see in the videos, but what facilitators who receive limited information on the setting might notice. Thus, by involving facilitators in a rating prior to using the video cases in facilitator PD, we ensure that the intended aim of the video cases, i.e., to facilitate teacher learning, can be pursued. In addition to validating for content, we asked the experts to suggest alternative moves and prompts that elicit facilitators’ noticing of teacher learning. With respect to designing the video cases for facilitator PD, we pursue the following research questions:

RQ 1: a) How do facilitators rate the representativeness and the quality of the video cases for use in facilitator PD courses?

b) When representativeness of the video cases with respect to SCK, PD-PCK, and facilitation moves was rated low, what reason do the facilitators provide for their estimation?

RQ 2: What facilitation moves do participants notice in the video cases and what alternative moves do they suggest as being beneficial for supporting teacher learning in PD settings?

RQ 3: What prompts do facilitators suggest to enhance noticing of facilitation moves in respect to the video cases?

## Method

In this chapter, we first describe the sample of our study. In particular, we outline participants’ range of experience in teaching mathematics and in facilitating PD courses for other mathematics teachers. Second, we elaborate on how the rating study was conducted. Third, we report on how we carried out the data analysis based on the theoretical frameworks and our design decisions.

### Sample

In order to answer our research questions, we conducted a rating study with eight PD facilitators. The participants of our study all hold a diploma in teaching mathematics, but recounted different degrees of practical experience as mathematics teachers and PD facilitators. Seven out of the eight participants have been working as mathematics teachers in primary school and high school for an average of 6 years, and one participant has been working as a high school mathematics teacher for 20 years. In regards to experiences in PD facilitation, participants have, on average, 6 years of experience in developing and implementing mathematics PD courses. Two participating facilitators reported facilitation experiences for 1 year, respectively 2 years, and two other participants had more than 8 years of experience leading PD courses for teachers. Six out of the eight participants are currently practicing PD facilitators. The remaining two participants were involved in planning and advising PD-related research at the time the study was conducted.

### Rating

Prior to the rating, we divided the eight participants of our study into two groups of four, Thereby, we chose a distribution ensuring that in each group, participants had a similar average of years of experience in facilitating mathematics PD. By so doing, we could reach our goal to assign each video case to participants with a variety of experience and were able to cover a broad spectrum of different perceptions. Each participant gained access to three of the selected six video cases, so that in sum, each case was watched and rated by four facilitators.

The rating process was structured by a rating protocol, including information on context and material used in each video case. What this information entailed was exemplarily outlined in chapter 3 for video case 1. Data was collected in open answer format according to the following categories: (1) mathematics content and its specific structure due to the learning goals (SCK); (2) teacher activities and crucial incidents of teachers’ artifact-based learning (PD-PCK); (3) perceived facilitation to advance teacher learning (facilitation moves); (4) alternative facilitation moves to support teacher learning (alternative moves); and (5) prompts to foster facilitators’ noticing of teacher learning.

The data collection for each video case comprised of the following steps*:* First, participants received detailed background information on the PD content of the video case: teacher learning goals, the mathematics tasks or results of teachers’ group work. Second, participants watched the whole video case for the first time and wrote down their answers to categories (1) to (4). After watching the scene a second time, participants provided their suggestions to category (5). Additionally, they evaluated the potential of each case for further use as a training tool during the course of a PD facilitator program. The *potential as a training tool* was operationalized as whether the video scene allows for intensive discussion about SCK, PD-PCK, and facilitation moves. On a four-point Likert scale ranging from 1 = strongly disagree to 4 = definitely agree, participants rated these categories. Scenes estimated below 3.0 will have further revision.

### Data analysis

Our data comprises both text documents of participants’ written answers to open questions concerning the five categories, and ratings on a Likert scale for evaluating the potential of the video cases for use in PD facilitator training programs. We conducted a qualitative content analysis in order to structure participants’ answers, applying deductive and inductive categorizations. First, we analyzed answers to questions from categories (1) and (2) to clarify particularly high and low ratings on the scale *potential as a training tool.* Participants’ answers to these questions gave us deeper insights in the observed SKC and PD-PCK aspects of each video case. Second, we analyzed the categories (3) and (4) concerning the perceived facilitation moves and the suggested alternative facilitation moves to foster teacher learning. For the analysis, we applied the facilitation move framework by van Es et al. (2014) in its modified version by González et al. (2016). Two researchers coded participants’ answers to categories (3) and (4) with respect to the different facilitation moves. The coding process was conducted within 1 week using the MAXQDA data analysis software. In general, the two researchers could mainly match participants’ described and suggested facilitation moves to those of the framework. For some answers, new categories of facilitation moves were developed. Disagreements in coding and the new categories were discussed among the two researchers until agreement was reached. In a similar manner, we analyzed the suggested noticing prompts of category (5) and distinguished the processes of selective attention, knowledge-based reasoning, and decision making in combination with the main categories SCK, PD-PCK and facilitation moves.

## Results and discussion

The aim of our study is to validate video cases as learning artifacts for facilitator PD. We report the results of our validation study according to our three research questions.

Research question 1.a) addresses facilitators’ estimation of the video cases representativeness for use in facilitator PD with respect to the three main content categories SCK, PD-PCK, and facilitation moves. Furthermore, we asked the facilitators to rate the quality of video and audio for each case. Table 5 shows the average rating for each of the six video cases.

**Table 5:** Representativeness and quality of the video scenes

Video-cases^a^		Representativeness of the video case^b^			Quality of the video case is sufficient^b^	
Number	Name	SCK	PD-PCK	Facilitation moves	Video	Audio
1	“Representing characteristics”	3.25	3.00	3.25	3.00	2.75
2	“Law of large numbers”	3.50	3.50	3.00	2.75	2.75
3	“Fitting representations”	3.25	3.50	3.50	3.00	3.00
4	“Symmetry of random events”	1.67	2.67	3.00	2.33	2.00
5	“The bonbon-task”	2.00	2.67	3.00	1.67	2.67
6	“The tower-task”	3.67	3.33	2.67	2.33	2.67

^a^The same group of four facilitators rated video cases 1, 2, and 3. The other four facilitators rated video cases 4, 5, and 6

^b^Ratings on a four-point Likert scale, ranging from 1 = strongly disagree to 4 = definitely agree

All video cases received an average rating of 2.50 or higher for the two categories PD-PCK and facilitation moves. Participants considered the video cases as representing vital opportunities to discuss aspects of PD-PCK and facilitation moves. Video case 6 received the lowest rating on facilitation moves. A possible reason for the medium rating might be the PD facilitator standing back and allowing a vivid discussion among the teachers for most of the video duration. In the following, we exemplarily present one rater’s description of the SCK aspect she noticed to be most relevant in video case 1:

The mathematics concepts involved in collecting statistical data require an open task such as the one the facilitator posed to the teachers. So that different questions are asked and different characteristics can be compared due to different illustrations. For some of the characteristics there are multiple options how to answer, eye-color for example. Others are more distinct and an interval is necessary. Facilitators have the opportunity to discuss these issues using the examples in the video (Rating B, video case 1, VS_DS1).

Regarding the video and audio quality, all cases that received a rating lower than 2.5 will be improved before final implementation.

Research question 1.b) focuses on facilitators’ specification of how the content categories are displayed in the video cases that perceived low to medium ratings. Only the video cases 4 and 5 fall into this category by showing low ratings of SCK. Video case 4, “Symmetry of random events”, shows a situation in which the PD facilitator asked the question whether selecting one element or four elements out of five objects is equal in terms of number. The participating teachers in the PD course at first could not answer the question and remained silent. The PD facilitator then tried to find a productive way to move on and left the question to be revisited at a later stage. Examining the qualitative data in terms of the raters’ explanations of the low score on SKC in this video case, we discovered the following: The facilitator in the video case decided to break the silence and to maintain an inquiry stance. These facilitation moves are dominant in this particular video case and therefore the SCK and PD-PCK aspects fade from the spotlight. One rater’s explanation alluded to this as follows:

The PD facilitator shows an example that illustrates the symmetry of random events. The problems of teachers’ understanding are prominent. The facilitator mentions the intent to later shed light on the mathematics concepts involved but that’s not shown in the video case (Rating D, video case VS_CP).

Although the SCK aspect is not fully pursued in video case 4, it is of course noticeable, and allows for in-depth discussion on how teachers’ learning can be fostered. We were able to observe a corresponding result considering the SCK aspect in video case 5. This case shows teachers of the PD course explaining different solutions to the following task: *In a bag there are three bonbons*: *a yellow one*, *a blue and a red one*. *You take two bonbons out of the bag. What is the probability of getting the red one*? *How does the probability change when adding more bonbons of different colors*? A discussion among the teachers occurred, focusing predominately on representations and how to use the task in classroom situations. The PD facilitator moderated the discussion, but stood back when it came to clarifying the mathematical concepts. This is what one of the facilitators referred to by stating:

It is very difficult to understand the SCK aspect in this video case because the discussion is all about suitable representations for the given task. But more, I would say, on a methodical level and not so much with respect to the mathematical concepts. The PD facilitator argues more from a pedagogical stance in her explanations so that the mathematics involved fades into the backlight (Rater E, video case VS_CO2).

Particularly for the SCK dimension, the qualitative data helped to clarify low ratings and provided hints regarding how to structure the video cases to guarantee a SCK focus.

Research question 2 addresses the facilitation moves participants in our study assigned to the video cases. We classified the facilitation moves according to the framework by González et al. (2016). Table 6 provides an overview of the distribution of facilitation moves with respect to the different categories.

**Table 6:** Facilitation moves noticed in the video cases

Category	Facilitation move	Video case						Total	% of total moves
		1	2	3	4	5	6		
Orienting the group to the task analysis	Launching	3	0	1	1	3	1	9	7.9
	Contextualizing	2	2	1	3	1	0	9	7.9
	Moving along^a^	0	1	0	4	0	0	5	4.4
Sustaining an inquiry stance	Highlighting	1	4	3	2	3	2	15	13.2
	Lifting up	4	1	3	2	3	2	15	13.2
	Pressing	2	0	1	1	0	0	4	3.5
	Clarifying	2	2	2	2	2	2	12	10.5
	Offering an explanation	0	1	2	2	1	1	7	6.1
	Countering	0	0	0	0	0	2	2	1.8
Maintaining a focus on the PD and the mathematics	Redirecting	3	0	1	2	0	3	9	7.9
	Pointing to evidence	2	0	0	0	0	0	2	1.8
	Connecting ideas	0	1	2	1	2	2	8	7.0
Supporting group collaboration	Standing back	2	0	1	0	4	4	11	9.6
	Distributing participation	0	0	0	2	0	0	2	1.8
	Validating participant ideas	1	0	1	0	0	3	5	4.4
Other moves	None	0	0	0	0	0	0	0	0.0
	Total number of moves	22	12	18	21	19	22	114	100.0

^a^The sum of the percentage of total moves does not equal 100% due to rounding up and down

The table shows the number of facilitation moves by video case and the percentage of total moves. The three moves that were noticed the most are *highlighting* (13.2%), *lifting up* (13.2%), and *clarifying* (10.5%). Together, these three moves make up more than a third (36.6%) of all incidents described by the participants of our study as facilitation moves.

Considering the four main categories of the facilitation move framework by van Es et al. (2014), modified by González et al. (2016), our results indicate that about half of the moves facilitators noticed in the video cases stem from the category *sustaining an inquiry stance* (48.3%). The other facilitation moves categories show significantly lower percentages: *orienting the group to the task analysis* (20.2%), *maintaining a focus on the PD and the mathematics* (16.7%), and *supporting group collaboration* (15.8%).

Additionally, the participants proposed alternative moves that can support teachers’ learning of mathematics in the specific scene (for an overview of all suggested alternative facilitation moves, see Table 7).

**Table 7:** Suggested alternative facilitation moves, benefiting teacher learning in PD

Category	Alternative facilitation moves	Video case						Total
		1	2	3	4	5	6	
Orienting the group to the task analysis	Launching	–	2	–	–	–	–	2
	Contextualizing	–	2	–	–	–	–	2
	Moving along	–	–	–	–	–	–	0
Sustaining an inquiry stance	Highlighting		–	–	–	–	–	0
	Lifting up	–	–	–	–	–	1	1
	Pressing	–	–	–	–	1	–	1
	Clarifying	–	–	–	3	–	–	3
	Offering an explanation	–	–	2	3	2	–	7
	Countering	–	–	1	–	–	1	2
Maintaining a focus on the PD and the mathematics	Redirecting	–	–	–	–	–	2	2
	Pointing to evidence	–	–	–	–	–	–	0
	Connecting ideas	–	–	–	–	1	1	2
	*Foregrounding SCK* ^a^	–	2	1	2	5	2	12
Supporting group collaboration	Standing back	2	2	1	1	1	–	7
	Distributing participation	2	–	1	1	2	–	7
	Validating participant ideas	1	–	–	–	–	–	0

^a^The text in italic describes new facilitation moves suggested by participants

Facilitators in our study suggested different alternative moves for each video case. The majority of these moves stem from the categories *sustaining an inquiry stance* (14), and *supporting group collaboration* (14). The other categories that are mentioned less frequently concern *orienting the group to the task analysis* four times and *maintaining a focus on the PD and the mathematics* four times as well. Furthermore, we were able to elicit additional moves that were not covered in the framework so far. We label these moves *foregrounding SCK* because they have a strong focus on the mathematical aspects of the PD course.

So far, we provided a detailed look into what *facilitation moves* participants of our study noticed in the video cases and what alternative moves they suggested. We presented the number of facilitation moves and provided the percentages of total moves. The reason behind this was to ensure that the selected six video cases, out of the many other possible sequences, hold the potential to exemplarily cover a wide range of possible learning opportunities for facilitating PD.

We will now exemplify what alternative moves the participants proposed for the existing categories, and will then turn our focus to the newly emerged category. First, we present two alternative moves to sustain an inquiry stance. In video case 3, the PD facilitator demonstrated the facilitation move *standing back* and one participant suggested the move of *offering an explanation* as an additional possibility:

For the second group, the facilitator delays a possible solution for an inappropriate representation. Without knowing the further progress of the PD, I am not sure if it might have been better to discuss the possible solution at this occasion as opposed to discussing it later. (Rating C, video case VS_DS3)

Another move for *sustaining an inquiry stance* was provided for video case 4 when teachers struggled with solving the task of whether one out of five equals four out of five:

As there was no answer given to the question how many possibilities one has to choose one objects out of five, I would have given more time to the teachers to think about the problem either on their own or by offering a discussion phase. (Rating F, video case VS_CP)

The additional move can be classified as *clarifying* the situation when teachers were confronted with a mathematical task they were not able to solve immediately. Regarding *supporting group collaboration*, one alternative move addressed the organization of a presentation phase. One participant suggested the following practice as alternative move for video case 1:

Instead of having the different groups of teachers present their results to the data collection task, the PD facilitator might use a different form of collaborative learning. For example, she might have the teachers prepare a short summary of their results in order to detect crucial aspects of mathematics themselves. (Rating B, video case VS_DS1)

The move can be classified as *supporting group collaboration*, as this is the prominent intention of the move. However, one can see that the reason behind the suggested practice is connected to promoting teacher learning. Thus, the generic aspect of applying a different method to organize collaborative learning draws on the intention to connect the teachers’ work more strongly to the mathematics involved. In terms of the theoretical framework we used, the alternative move can be assigned to *validating participant ideas*. In the same main category, addressing the sub-category of *standing back*, one participant offered the subsequent alternative move:

The PD facilitator in this video case seems to be highly skilled in keeping the attention on teachers’ presentations, and taking notes at the same time. She is also able to remember teachers’ statements in detail. As an alternative, I suggest that she could ask teachers of the non-presenting groups to list crucial aspects for her as well. (Rating B, video case VS_DS1)

The suggestion of involving other teachers, who are not actively presenting, would allow the facilitator to stand back and to concentrate on the presentation of results, and would simultaneously activate rather silent teachers to engage in the mathematics task. Thus, as a result of the move, the facilitator would also contribute to the category of *distributing participation*, but as this aim was not in the main focus of the proposal, we opted for coding the category with *standing back*.

In addition to the theoretically derived facilitation moves, we were able to identify moves that are not comprehensively covered in the framework thus far and that aim at *foregrounding SCK*. This category is strongly related to the ultimate goal of strengthening teachers’ mathematics learning and is thus directly connected to helping teachers attain the mathematical learning goals. The following examples illustrate what we consider as moves to especially foreground aspects of SCK:

To illustrate the law of large numbers I would have used a different set of trial numbers like multiples of 36 for instance 72, 360 and 36000. This makes it easier to understand the distribution of the relative frequency. (Rating B, video case VS_DS1)

It would have been helpful to prepare some inappropriate representations to focus aspects such as missing labeling, skewed sample or missing zero (only the first aspect is mentioned in the video case). It might also be helpful to present different representations of the same data. (Rating C, video case VS_DS3)

In these statements, the two participants of our study provided some suggestions for focusing on additional aspects of SCK. Thus, teachers would get the chance to deepen their mathematical knowledge. In the same vein, the subsequent suggestions for alternative moves show how SCK aspects can be utilized as starting point to deepen PD-PCK:

When the teacher stated “that it is logical for her that the result is 2/3”, I would have asked how one can explain that to students in the classroom. Thereby, one would have provoked explanations by the other participants as well. (Rating E, video case VS_CO1)

When the teacher explained “to draw them all at once, I get that I need to write down pairs,” the facilitator could have asked if this viewpoint is sufficient or if it would cause problems. (Rating F, video case VS_CO1)

The newly emerged sub-category *foregrounding SCK* thus enriches the main category of *maintaining a focus on the PD and the mathematics*.

These examples enable a deeper look into experienced facilitators’ thinking processes on how to act as a PD facilitator in a challenging situation. As the six video cases stand out for many other possible scenes, we thereby ensure that the chosen cases provide enough variance with respect to discussing this content category. In the discussion (chapter 6), we will further elaborate on how these different types of information were used to compile the final video cases.

With research question 3, we turn our attention towards designing video-based PD to allow prospective facilitators to enhance their noticing of crucial incidents of teacher learning in PD. Thus, we investigated facilitators’ perception of crucial prompts and questions in order to enhance the noticing of facilitation moves in the video cases. Our rating study shows that the suggested prompts, in general, address the categories *SCK* or *PD*-*PCK* and *facilitation moves*. Additionally, we were able to differentiate the prompts by applying the three processes of noticing: *selective attention*, *knowledge*-*based reasoning*, and *decision*-*making*. Table 8 presents an overview of facilitators’ suggested prompts per video case structured by content and noticing processes.

**Table 8:** Distribution of suggested prompts to the content and noticing categories per video case

Content category	Noticing prompts	Video case						Total
		1	2	3	4	5	6	
SCK/PD-PCK	Selective attention	1	0	1	0	0	0	2
	Knowledge-based reasoning	3	4	4	3	2	6	22
	Decision-making	0	4	2	0	4	4	14
Facilitation moves	Selective attention	1	0	0	2	1	0	4
	Knowledge-based reasoning	3	1	2	6	8	2	22
	Decision-making	9	4	4	5	4	5	31
Other	Selective attention	0	0	0	0	0	0	0
	Knowledge-based reasoning	2	0	0	1	0	0	3
	Decision-making	0	0	0	0	0	0	0
Total		19	13	13	17	19	17	98

As the suggested noticing prompts did not address SCK or PD-PCK separately from each other, we coded the two categories together. In sum, facilitators suggested 38 prompts focusing on both SCK and PD-PCK for the 6 video cases. The remaining 57 prompts concerned facilitation moves, and only 3 could not be assigned to any of these categories. In total, 38.8% of all prompts were related to SCK/PD-PCK while 58.2% addressed facilitation moves. The distribution among the 6 cases was balanced: at the lowest, 13 prompts were suggested for video cases 2 and 3, and at the highest, cases 1 and 5 received 19 suggestions for noticing questions. Only 6 questions addressed *selective attention*, 47 of them pertained to *knowledge*-*based reasoning* and 45 were related to *decision*-*making.* The amount of suggested noticing prompts demonstrates that the video cases contain enough variability to allow for noticing incidents of the three content categories. In our approach, the category of *selective attention* has minor relevance. As the video cases will be used in facilitator-led facilitator PD, the facilitator will pose noticing prompts to deliberately focus selective attention on specific incidents.

In the next section, we will exemplify what questions the facilitators suggested to the different categories of SCK/PD-PCK and facilitation moves as well as the two noticing categories *knowledge*-*based reasoning* and *decision*-*making*. The suggested prompts differ in respect to their nature. Some of them are rather generic and might be applied to different scenes, and others are specific and closely related to the learning of mathematics displayed in the video case.

In the final design of the video cases, we will consider the suggested prompts to redirect facilitators’ noticing to the essential content categories (Table 9).

**Table 9:** Suggested noticing prompts regarding knowledge-based reasoning and decision-making for the categories SCK/PD-PCK and facilitation moves

	Knowledge-based reasoning	Decision-making
SCK/PD-PCK	What problem does the contribution of the teacher (refers to a building with three entrances) adds to the discussion? How is this related to the teacher learning goals?	In case the mentioning of teachers would not have occurred, would you have brought in this possible form of representation on your own? If yes, for what reasons?
	For what mathematical reason did the facilitator not interrupt the lengthily discussion of the teachers?	Would you collect the different representation forms contributed by the teachers in a different way? Why?
	Why is the example of 3! not selected in a good way with respect to teachers’ misconceptions?	
	What PD-PCK aspects might help to resolve teachers’ misconceptions?	
	Is the discussion on the different representation forms sufficient with respect to teaching this subject in the classroom?	
Facilitation moves	Why do you think has the facilitator decided to have the teachers solve the task for the students themselves?	Which alternative moves would you have performed instead of the one shown by the facilitator?
	Why did the facilitator choose to let only one teacher present the result?	How could you foster that the teachers reflect more intensively the teaching perspective?
		Does the facilitator act correctly when letting the discussion continue?
		Are the shown misconceptions of teachers treated sufficiently?

In our study, we reported on designing video cases for use in facilitator PD that show crucial incidents of teacher learning, and allow for probing how to adequately support this learning. Focusing on preparing facilitators for their crucial role has received much attention in recent years and different frameworks emerged, capturing the *knowing* and *acting* of facilitation (Tekkumru-Kisa and Stein 2017). Against the backdrop of such research findings and theoretical contributions, the approach of our validation study, which involved facilitators with variant levels of experience, was twofold: First, we explored whether the video cases show incidents of SCK, PD-PCK, and facilitation moves related to teacher learning. Second, we confirmed that the video cases possess enough variance to reflect upon the three content categories.

Our first research question addressed whether the representativeness and the quality of the video cases are sufficient for using them in facilitator PD courses. As the purpose of compiling the video cases was to discuss how to support teacher learning of mathematics, we validated them with respect to the three categories of SCK, PD-PCK, and facilitation moves. These three categories represent fundamental perspectives on how to analyze the core of facilitators’ knowledge and skills to effectively perform in supporting mathematics teachers’ learning in PD. Additionally, we carefully considered low ratings to ensure that the chosen video cases cover the three categories comprehensively. Overall, the estimations for the video cases were sufficient to good. Low ratings only occurred for the SCK category. We explored the reasons behind the estimations, and on this basis, we can now decide how to keep the focus on SCK by adding noticing prompts.

The second research question focused on classifying the facilitation moves shown in the video cases. In sum, the participants of our study noticed 114 facilitation moves. These could be assigned to the categories taken from the González et al. (2016) framework: *orienting the group the task analysis*, *sustaining an inquiry stance*, *maintaining a focus on the PD and the mathematics*, and *supporting group collaboration.* About half of the facilitation moves were categorized to *sustaining an inquiry stance*, indicating that the chosen video cases come close to showing processes of teacher learning in PD situations. Besides this focus, participants of our study frequently noticed facilitation moves out of all main and sub-categories. The video cases thus present vital opportunities to discuss the moves and decisions of a PD facilitator in action, especially those that concentrate on attaining the mathematics learning goals. These findings go in line with our expectations and offer a promising approach to involve the video cases in future qualification programs for prospective facilitators.

The six video cases predominately show situations of a PD course in which the PD facilitator interacts with the teachers to foster their mathematics learning. Thus it is not surprising that the moves our participants noticed are concerned with attending to crucial teachers’ ideas (*highlighting*), identifying important teachers’ contributions for fruitful discussions (*lifting up*) as well as restating and clarifying the common understanding of teachers’ ideas (clarifying). On the other hand, it stood out to us that the facilitation move *standing back* was noticed quite often as well, displaying a crucial move that the facilitator in the video performs often to keep participants involved in a vibrant mutual exchange of thoughts. The video cases also allow for discussing alternative moves as the results of our validation study show. In sum, 48 alternative facilitation moves were suggested; thus the video cases possess rich variance with respect to discussing different actions that facilitators might perform. Twelve out of these moves could be assigned a newly emerging category that focuses on foregrounding SCK aspects during the course of the PD. As the participants in our study were explicitly asked to suggest facilitation moves to foster substantial teacher learning, it is not surprising that the main category of *maintaining a focus on the PD and the mathematics* was further developed. Summing up, we can conclude that the six video cases exemplarily allow for discussing a rich variety of facilitation moves.

The third research question was concerned with exploring how the video cases can be used to compile structured facilitator PD. That is, when the video cases will be used in a facilitator PD, noticing prompts will enrich the scenes. As research findings confirm, such structured video clubs (cf. van Es and Sherin 2006; Beisiegel et al. 2018) help participants to focus on specific aspects and avoid discussions on a general level. In correspondence with the first two research questions, the noticing prompts elaborate on revealing SCK/PD-PCK aspects and involve knowledge-based reasoning and decision-making. In sum, the participants of our study suggested 98 prompts from which almost half of them could be assigned to the 2 content categories. The quality of the suggested prompts differed from being rather general to specific.

## Conclusions

So far, we could reach the main aim of our validation study with respect to the content of the video cases: All six video cases present valid examples of facilitators fostering teacher learning and can be used as artifacts to foster PD facilitators’ learning. The suggested alternative facilitation moves and noticing prompts specified the proposed intention of discussing the video cases. Based on our results, we will be able to finally compile the video cases and to enrich them with respect to the noticing categories that will play a prominent role in the PD for prospective facilitators.

In reflecting upon our study, we encountered several challenges, which on the one hand are crucial for our future work, but on the other hand extend to the research on PD facilitation in general. The first challenge lies within bridging different frameworks and conceptualizations to describe what knowledge and skills PD facilitators need to foster mathematics teachers’ learning. Applying the facilitation move framework by van Es et al. (2014) and González et al. (2016) was helpful to capture the facilitator skills from a generic perspective, but to reveal the specificity of the moves with respect to the mathematics involved, we needed a more content-related approach. As the ratings on SCK received the lowest results, we will strengthen the focus on this category.

The second challenge concerns our approach to develop and validate video cases from authentic PD courses with the purpose of qualifying prospective PD facilitators. From a methodical perspective, we raised the question what *evidence of validity* these video cases need to provide reliable learning opportunities for prospective PD facilitators. Even though our results show sufficient ratings in terms of SCK, PD-PCK, or possible facilitation moves involved, we could show that specific prompts are needed to unlock the full potential of the video cases as learning incidents. This observation is in line with research on facilitating video-based discussion (cf. Borko et al. 2014a, b; Borko et al. 2017).

Although our results are of course bound to the six video cases we selected, our approach can easily be transferred to other contexts. The procedure of compiling the video cases and validating them is in line with the most prominent research findings on designing facilitator PD. We exemplarily showed how empirical evidence could be used to prepare facilitator PD.

## Abbreviations

PCK: Pedagogical content knowledge
PD: Professional development
SCK: Specialized content knowledge
